# Supplementary material for: Isolation, Maintenance and Differentiation of Primary Tracheal Basal Cells from Adult Rhesus Macaque
Source: Methods Protoc. 2019 Oct 1;2(4):79. doi: 10.3390/mps2040079 (PMC6961120; doi:10.3390/mps2040079)
Supplement: Supplementary file 1 [file mps-02-00079-s001.zip › Engler_MDPI_Supplementary.docx]

Supplementary Materials and Methods

Supplementary Materials

Table 3: Supplemental Materials S1

| **Material** | **Company** | **Reference Number** |
| --- | --- | --- |
| **Chemicals** |  |  |
| Bovine Collagenase, PureCol | Advanced Biomatrix | 5005-100ML |
| DNAse | Sigma | DN25-100MG |
| Protease | Sigma | P5147-100MG |
| Penicillin Streptomycin | Gibco | 15140-122 |
| Amphotericin B/Fungizone | ThermoFisher | 15-290-018 |
| Primocine | Invivogen | Ant-pm-2 |
| DMEM/F12 | Gibco | 11330-032 |
| Phosphate-buffered saline (PBS) | Gibco | 14190-144 |
| Phosphate-buffered saline (containing CaCl and MgCl) | Gibco | 14040-133 |
| 0.25% Tyrpsin | Gibco | 25200-056 |
| Fetal Bovine Serum | HyClone, GELifeScience | SH30071.03 |
| Fetal Donkey Serum | JacksonImmuno | 017-000-121 |
| Bovine Serum Albumin | Fisher Bioreagents | BB1600-100 |
| Paraformaldehyde | Fisher Chemical | 04042-500 |
| 3D Matrigel, Matrigel Matrix Basement Membrane | Corning | 356231 |
| Rho Kinase Inhibitor Y-27632 | Tocris | 1254 |
| **Antibodies** |  |  |
| Cytokeratin5 | Covance | PRB160-P |
| Trp63 | Genetex | GTX102425 |
| Trp63 | Santa Cruz | SC-25268 |
| Cytokeratin14 | ThermoScientific | MS115P1 |
| Acetylated Tubulin | Sigma | T7451 |
| Click-It EdU Detection | Invitrogen | C10637 |
| Höchst 33342 | Invitrogen | H1399 |
| Calcein Blue | Invitrogen | C1429 |
| **Plastic Ware** |  |  |
| 10 cm dish | Falcon/Corning | 353003 |
| Multiwell Chamber Slides | Thermo Fisher | 177402 |
| 24-well plates | Corning/Costar | 3524 |
| Cell Culture Insert 24well format, 0.4 µm | Falcon | 353095 |
| Sterile Syringe Filter, 0.2 µm | Corning | 431222 |
| 5mL Polystyrene Round bottom tube with cell strainer cap | Falcon/Corning | 352235 |
| **Equipment** |  |  |
| Hoods | Labconco | Logic+ Purifier |
| Centrifuge | Thermo Scientific | Sorvall ST8R |
| Automated Cell Counter | Logos Biosystems | Luna II |

Supplementary Methods

Immunocytochemistry

Cells on insert or glass cover slip were fixed for 5-1 minutes in 4% PFA in PBS and washed 3 times thereafter. Cells were blocked for 30 minutes, using 10% normal donkey serum (NDS), 4% bovine serum albumin (BSA) and 0.5% TritonX. Blocking solution was replaced with 2.5% NDS and 1% BSA, containing primary antibodies. Primary antibodies were incubated overnight at 4C. Cells were washed using PBS-T (0.1% TritonX) and incubated in 5% NDS, 2% BSA, 0.25% TritonX containing secondary antibodies for 1 hour, at RTC. Cells were washed using PBS-T (0.1% TritonX) and incubated for 5 minutes with Höchst, nuclear dye. Cells were washed once more with PBS and remaining PBS was diluted with ddH2O. Insert or glass coverslip were immediately mounted with FluorSave (Millipore) after ddH2O wash. Slides were dried for 24 hours before fluorescent analysis.

FACS Analysis

Cells were dissociated from plate and resuspended in 500 µL PBS in a 15 mL conical tube. 500 µL of 8% PFA in PBS was added to obtain a 4% fixation solution. Cells were fixed for 5 minutes. 14 mL of PBS were added to the fixation solution and cells were spun down at 300g, 5 minutes. Supernatant was removed and pellet was resuspended in 1 mL of PBS. For permeabilization and cell cycle analysis using nuclear dyes such as Höchst, 4′,6-diamidino-2-phenylindole (DAPI) or Propidium Iodide (PI) cells need to be postfixed using ethanol. Ethanol is added in a dropwise fashion to a final concentration on 90% ethanol. Cells need to be permanently agitated while ethanol is added, using a vortex at low speed. Cells are put to -20°C for 30 minutes (or maximum for 5 day storage). For staining, ethanol needs to be washed of cells. Tube needs to be acclimatized to room temperature for 20 minutes, before cells are spun down at 300g, 5 minutes. Cell pellet is washed twice with PBS, with intermittent centrifugation steps. Cells can then be resuspended in directly conjugated FACS antibodies, nuclear dyes or primary antibodies followed by secondary antibodies. For all stainings 1% BSA in PBS (containing CaCl and MgCl) was used.

EdU Incorporation for Cell Cycle Analysis

Cells were cultured in Pneumacult-Ex or Pneumacult-ALI according to protocol. For cell cycle analysis EdU was added to fresh medium to a final concentration of 10 µM and cells were incubated with medium containing EdU for 2 hours for S-Phase analysis. After 2 hours, cells were washed 2x with PBS and fixed for 5 minutes in 4% PFA. Stainings were performed as usual, keeping the Far Red channel empty for EdU detection. For EdU detection the Click-It protocol was used according to manufacturer’s guidelines.
